# Intrauterine Reprogramming of the Polycystic Ovary Syndrome: Evidence from a Pilot Study of Cord Blood Global Methylation Analysis

**DOI:** 10.3389/fendo.2017.00352

**Published:** 2017-12-18

**Authors:** Luca Lambertini, Shira Rebecca Saul, Alan B. Copperman, Sara Salehi Hammerstad, Zhengzi Yi, Weijia Zhang, Yaron Tomer, Nathan Kase

**Affiliations:** ^1^Department of Environmental Medicine and Public Health, Icahn School of Medicine at Mount Sinai, New York, NY, United States; ^2^Diabetes Obesity and Metabolism Institute, Icahn School of Medicine at Mount Sinai, New York, NY, United States; ^3^Department of Obstetrics, Gynecology and Reproductive Science, Icahn School of Medicine at Mount Sinai, New York, NY, United States; ^4^Division of Endocrinology, Diabetes and Bone Diseases, Icahn School of Medicine at Mount Sinai, New York, NY, United States; ^5^Division of Endocrinology, James J. Peters Veterans Affairs Medical Center, Bronx, NY, United States; ^6^Department of Pediatrics, Oslo University Hospital, Ullevål, Oslo, Norway; ^7^Department of Endocrinology, Morbid Obesity and Preventive Medicine, Oslo University Hospital, Aker, Oslo, Norway; ^8^Institute for Personalized Medicine, Icahn School of Medicine at Mount Sinai, New York, NY, United States; ^9^Department of Medicine, Albert Einstein College of Medicine, Montefiore Medical Center, Bronx, NY, United States

**Keywords:** polycystic ovary syndrome, epigenetics, pregnancy, metabolic syndrome, diabetes

## Abstract

Polycystic ovary syndrome (PCOS) affects 5–15% of women. PCOS is a heterogeneous disorder displaying endocrine, metabolic, and reproductive dysfunction and cardiovascular risk manifestations. Evidence of heritability exists, but only a portion of the genetic transmission has been identified by genome-wide association studies and linkage studies, suggesting epigenetic phenomena may play a role. Evidence implicates intrauterine influences in the genesis of PCOS. This was a pilot study that aimed at identifying an epigenetic PCOS reprogramming signature by profiling the methylation of the DNA extracted from umbilical cord blood (UCB) from 12 subjects undergoing *in vitro* fertilization. Six subjects were anovulatory PCOS women diagnosed by Rotterdam criteria and six ovulatory non-PCOS women matched for age and body mass index. UCB was collected at delivery of the placenta; the DNA was extracted and submitted to methylation analysis. A differential methylation picture of prevalent hypomethylation affecting 918 genes was detected. Of these, 595 genes (64.8%) carried single or multiple hypomethylated CpG dinucleotides and 323 genes (35.2%) single or multiple hypermethylated CpG dinucleotides. The Ingenuity Pathway Analysis (IPA) online platform enlisted 908 of the 918 input genes and clustered 794 of them into 21 gene networks. Key features of the primary networks scored by IPA included carbohydrate and lipid metabolism, neurotransmitter signaling, cardiovascular system development and function, glycosaminoglycan signaling regulation and control of amino acid biosynthesis. Central to the network activities were genes controlling hormonal regulation (*ESR1*), mitochondrial activity (*APP, PARK2*), and glucose metabolism (*INS*). Regulatory pathways such as G-protein coupled receptor signaling, inositol metabolism, and inflammatory response were also highlighted. These data suggested the existence of a putative “PCOS epigenomic superpathway” with three main components: glucotoxic, lipotoxic, and inflammatory. If our results are confirmed, they hint at an epigenetic at risk PCOS “signature” may thus exist that may be identifiable at birth. Additional studies are needed to confirm the results of this pilot study.

## Introduction

The Polycystic Ovary Syndrome (PCOS) is one of the most common human endocrine/reproductive/metabolic disorders; depending on the definition applied (1–3), PCOS affects 5–15% of women (4). Its mature form is characterized by hyperandrogenicity, ovulatory and menstrual dysfunction, polycystic ovary morphology, and distorted gonadotropin secretory activity (5). PCOS patients manifest the endocrine and metabolic burdens associated with the metabolic syndrome, including selective tissue insulin resistance, dyslipidemia, and endothelial dysfunction (6). These burdens are amplified by the compounding influence of generalized obesity, particularly evidenced by central visceral adipose tissue accumulation (5). Other features include increased frequency of endometrial cancer and psychiatric disorders (7).

The strong heritability of PCOS is supported by twin (8) and genetic studies (9) as well as familial clustering of phenotypic features (6) which is observed in 20–40% of patients (6, 10). The search for “culpable” PCOS genes has been extensively reviewed by Dunaif (9) and Azziz (4). Although GWAS have identified 16 putative PCOS susceptibility genes, several of which have been confirmed in large, ethnically diverse populations, their contribution represents a small portion (<10%) of the total transmission of the syndrome (4). Furthermore, despite the strong clinical association of PCOS with type 2 diabetes and the metabolic syndrome, none of the major genes associated with type 2 diabetes mellitus or obesity are significantly associated with PCOS (9).

Since genetic factors cannot explain the majority of the risk for PCOS, other mechanisms must play a role. One potential mechanism that could yield the same phenotypic heritability as genetics is an epigenetic process *via* an adverse intrauterine environment (6, 11, 12). Supporting an epigenetic influence during pregnancy are studies by Rosenfield (13) showing an association between initial maternal weight, weight gain in pregnancy, and delivery of babies who later develop PCOS. A putative influence of the intrauterine environment is further supported by primate studies in which administration of testosterone in mid-gestation induced the PCOS phenotype in female progeny (6). However, the prevalence of PCOS in women from opposite-sex twin pairs in which acquisition of testosterone from the male co-twin could adversely affect the female co-twin, is not different than that in same sex twin pairs (14), demonstrating that high testosterone levels during pregnancy could not be the sole explanation for the transmission of the PCOS phenotype to the baby. Indeed, second generation offspring from pregnancies not administered androgen also develop similar PCOS phenotypic manifestations, suggesting an intrauterine epigenetic program independent of androgens and can be carried forward transgenerationally (15).

Given evidence from twin studies in which a shared intrauterine environment exposure exists, we hypothesized that PCOS during pregnancy induces fetal epigenetic reprogramming resulting from intrauterine conditions imposed by three factors: (a) the pre-pregnancy metabolic, endocrine, and vascular changes of the PCOS mother; compounded by (b) the prevailing physiologic changes of pregnancy; and (c) the acute pathologic dysfunctions (hypertension, gestational diabetes) associated with pregnancy in PCOS patients. To test this hypothesis, we compared the global methylation patterns in umbilical cord blood (UCB) of neonates delivered from PCOS and non-PCOS women. Although the results of this pilot study must be considered preliminary, our data suggest the existence of a “PCOS epigenomic superpathway”—a PCOS epigenetic signature—involving 10 differentially methylated gene networks governing lipid, carbohydrate metabolism, and inflammation/immunologic systems.

## Materials and Methods

### Patients

This was a single-center, prospective cohort study designed and conducted in accordance with the principles expressed in the Helsinki Declaration. To avoid selection bias, subjects were enrolled sequentially from patients attending our clinics. All subjects provided written informed consent before enrollment. The protocol was approved by the Icahn School of Medicine at Mount Sinai Institutional Review Board (IRB) (HS#: 12-00714, GCO#: 12-1367). Consenting subjects received a full infertility work-up including measurement of serum anti-Müllerian hormone (AMH), FSH, and LH concentrations and transvaginal ultrasound (performed on day 3 of menstruation), and were deemed appropriate candidates for *in vitro* fertilization. Patients with irregular menstrual cycles and/or signs of hyperandrogenism had measurement of androgen levels and clinical evaluation of hirsutism through the Ferriman–Gallwey score. Twelve patients were recruited; six with PCOS and six without PCOS. The diagnosis of PCOS in study patients was based on the 2003 Rotterdam consensus criteria. Patients were diagnosed with PCOS by meeting two of the following three criteria: oligo/anovulation, hyperandrogenism (either clinical or biochemical), and sonographic evidence of polycystic ovaries. Clinical hyperandrogenism was defined clinically by the presence of hirsutism, alopecia, and/or acne, or subclinically by increased levels of serum testosterone and/or dehydroepiandrosterone sulfate. Polycystic ovaries were diagnosed by pelvic sonography according to the Rotterdam conference criteria. Patients who met Rotterdam criteria for PCOS were identified through a natural language processing query of the electronic medical record data base. They were approached in accordance with approved IRB protocols, and all signed informed consent for participation in the study. There were no exclusion criteria. The control group included ovulatory women matched for age and BMI without PCOS features and delivered a full-term singleton over the same time period (Table 1).

**Table 1:** Patient characteristics.

Variables	Control (*n* = 6)	Polycystic ovary syndrome (*n* = 6)	*p*-Value^a^
Age	40.1 ± 6.2 (33.7–47.9)	33.9 ± 2.0 (30.5–35.6)	0.07
BMI	26.2 ± 4.3 (20.9–31.1)	25.9 ± 5.6 (20.7–33.5)	0.90
Anti-Müllerian hormone	2.0 ± 0.3 (1.8–2.2)	6.8 ± 2.0 (4.5–8.3)	0.04^b^
FSH	5.8 ± 3.3 (2.1–9.8)	5.6 ± 2.5 (2.3–8.2)	0.20
LH^c^	3.5 ± 1.7 (1.1–5.3)	6.7 ± 3.4 (4.2–13.2)	0.10
E2^c^	47.5 ± 17.9 (33.2–72.8)	43.9 ± 20.2 (23.2–74.1)	0.78
P4^c^	0.6 ± 0.5 (0.2 ± 1.2)	0.3 ± 0.1 (0.2–0.4)	0.46
FSH/LH ratio^c^	1.8 ± 0.7	0.9 ± 0.5	0.30
Antral follicle count^c^^,^^d^	6.8 ± 2.2 (4–9)	29.2 ± 13.7 (18–52)	0.02^b^
Menstrual cycle abnormalities	Yes = 0/no = 6	Yes = 6/no = 0	–
Acne/hirsutism	Yes = 0/no = 6	Yes = 6/no = 0	–
Infant gender	Females = 4/males = 2	Females = 2/males = 4	–

*^a^The Student’s t-test was used to compare means of two groups except for the antral follicle count*.

*^b^p < 0.05*.

*^c^Obtained on day 3*.

*^d^Chi-square test was used to compare between proportions. When more than 20% of the expected counts were less than 5, Fisher’s exact test was applied*.

### Sample Collection and Processing

Umbilical cord blood was collected at delivery of the placenta in Purple Top BD Vacutainers (Franklin Lakes, NJ, USA), thoroughly mixed and aliquoted in 2 ml cryovials (Thermo Fisher Scientific, Waltham, MA, USA), snap frozen in liquid nitrogen and stored at −80°C.

The CXT 350 frozen sample aliquotter (CryoXtract, Woburn, MA, USA) was used to retrieve tissue subaliquots for downstream applications to prevent the thawing of the full UCB aliquots. DNA was extracted using the Maxwell 16 automated DNA/RNA extraction equipment (Promega, Madison, WI, USA) and quantified with Nanodrop spectrophotometer (Thermo Electron North America, Madison, WI, USA).

### Methylation Analysis

DNA samples were subjected to methylation analysis using the Illumina Infinium HumanMethylation450 BeadChip Kit (Illumina, San Diego, CA, USA) at the University of Cincinnati Genomics, Epigenomics and Sequencing Core (GESC). Native DNA was sent from our labs to GESC. GESC personnel were blinded about the samples’ diagnosis. Samples were treated following the HumanMethylation450 BeadChip consolidated protocol. Briefly, native DNA was bisulfite treated using the EZ-96 DNA Methylation-Gold Kit according to the manufacturer’s protocol (Zymo Research, Irvine, CA, USA). Bisulfite-treated DNA was then hybridized to HumanMethylation450 BeadChips that were later scanned. The intensities of the images were extracted using the GenomeStudio (v.2011.1), “Methylation Module” (1.9.0) software (Illumina, San Diego, CA, USA), which normalizes within-sample data using different internal controls that are present on the HumanMethylation 450 BeadChip and internal background probes.

Processed data were sent back to our lab for analysis. Probes with a low signal (detection *p* > 0.05) across all samples were excluded from analysis. For each CpG dinucleotide, we calculated a Beta-value representing the ratio of the methylated probe intensity and the overall intensity (sum of methylated and unmethylated probe intensities). Beta-values spans between 0 and 1, or 0 and 100%. Following a consolidated procedure, we transformed the Beta-values into *M* values using the following equation:

$$M={\text{log}}_{2}\left[\text{Beta}/(1-\text{Beta})\right].$$

This transformation provides a straightforward method for using the Beta-value statistic and obtaining the unique statistical properties of the *M*-value which are not compressed in the low and high range, provide better insights into the distribution of methylation, and can be better handled by statistical tools (16). To identify differentially methylated genes between UCB DNA samples taken from PCOS pregnancies vs controls, paired Linear Models for Microarray (LIMMA) test was performed between PCOS and controls. LIMMA is a statistical tool that was designed for gene expression analysis and it has found usage outside this area. It performs parallel linear regression analyses using an empirical Bayes approach to shrink the estimated sample variances toward a pooled estimate. This approach produces robust/stable estimates, especially for small sample size (17).

Because of the limited number of subjects, the analysis was focused on differentially methylated CpG dinucleotides with a minimum twofold methylation change obtained as:

$$\text{Fold change}=2^{\left|(\text{AVG}{\left(M\right)}_{\text{Cases}}-\text{AVG}{\left(M\right)}_{\text{Controls}})\right|}.$$

For all genes carrying multiple differentially methylated CpG dinucleotides, the *M* methylation value was averaged in order to score the gene overall methylation level. Some 23 genes carried both hyper- and hypomethylated CpG dinucleotides; by averaging the CpG dinucleotides *M* methylation values we obtained 12 genes with an overall hypermethylated profile and 11 with a hypomethylated profile (Tables S1, S2, and S7 in Supplementary Material).

Additionally, because of the asymmetric distribution of sexes of the babies in the control and PCOS groups (Table 1), in our primary analysis we removed all sex-specific differentially methylated CpG dinucleotides. A subsidiary analysis was also performed to determine if any residual gender-specific difference in methylation profiles of male and female PCOS offspring vs their control gender counterparts existed.

### Network and Pathway Analyses

Network and pathway analysis was conducted using the Ingenuity Pathway Analysis (IPA) engine for the analysis of “omics data” (http://www.ingenuity.com). The standard setup for network analysis provided by the IPA core analysis was employed with one exception; because of the confidence provided by the large amount of input genes, the molecules per network parameter was set at 140.

All genes carrying single and multiple, concordant and discordant differentially methylated CpG dinucleotides were used for the IPA analysis. A total of 918 unique genes were thus used (see Tables S1, S2, and S5 in Supplementary Material for their differential methylation status). Genes were fed to IPA complete of their differential methylation *M* values as:

$$\text{Differential }M\text{ Methylation Value}=(\text{AVG}{\left(M\right)}_{\text{Cases}}-\text{AVG}{\left(M\right)}_{\text{Controls}}).$$

Only significant genes and pathways identified by employing the built-in right-tailed Fisher’s exact test were used for the network-specific canonical pathway analysis and constructing the PCOS “superpathway.”

## Results

### Mapping Differentially Methylated CpG Regions

**Figure 1:**
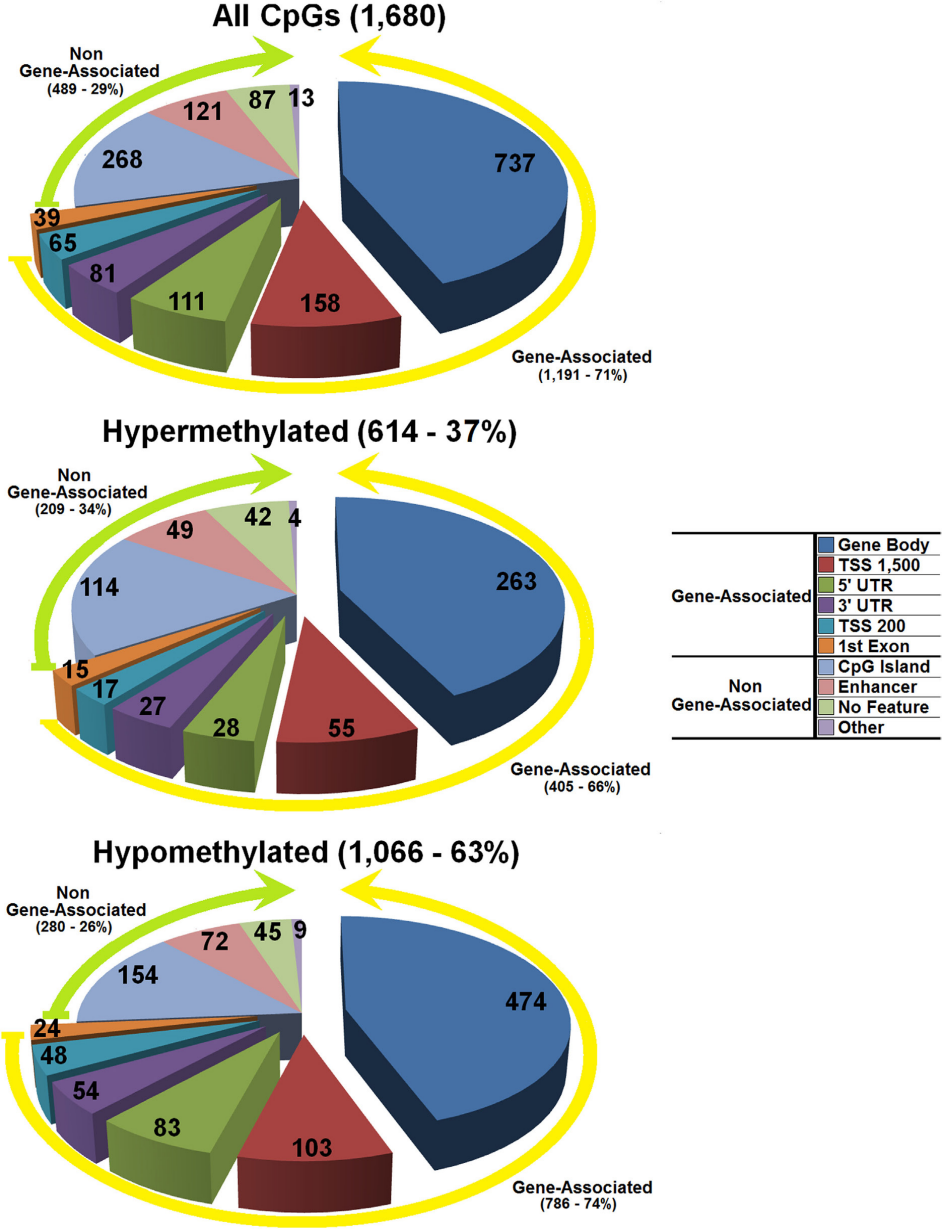
Distribution of the differentially methylated CpG dinucleotides in the umbilical cord blood from offspring of polycystic ovary syndrome mothers vs controls with a minimum twofold methylation change. Top panel all differentially methylated CpG dinucleotides; mid panel hypermethylated CpG dinucleotides; bottom panel hypomethylated CpG dinucleotides. The exploded portion of the pie charts reports the classification of those differentially methylated CpG dinucleotides that map in or near know genes. The other portion of the pie chart reports the main characteristics of the differentially methylated CpG dinucleotides that do not map in or near known genes.

A total of 614 hypermethylated and 1,066 hypomethylated CpG dinucleotides were identified (Figure 1; Tables S1 and S2 in Supplementary Material). Some 209 of 614 hypermethylated and 280 of 1,066 hypomethylated CpG dinucleotides mapped to genomic regions not associated with any known gene (Table S3 in Supplementary Material). These CpG dinucleotides showed a distribution strongly associated with the length of the chromosome they map within (Spearman’s rho for non-parametric bivariate correlation: hypomethylated CpG dinucleotides = 0.720; *p*-value < 0.001—hypermethylated CpG dinucleotides = 0.739; *p*-value < 0.001) (Table S4 in Supplementary Material).

The remaining 405 of 614 hypermethylated CpG dinucleotides mapped in or near 335 genomic tracks banked in the UCSC genome browser (https://genome.ucsc.edu/cgi-bin/hgGateway—GRCh37/hg19 Assembly), and 786 of 1,066 hypomethylated CpG dinucleotides mapped in or near 606 genomic tracks banked in the same database (Figure 1; Tables S5 and S6 in Supplementary Material). Out of a total of 941 banked genomic tracks, 918 referred to unique genes; of these, 895 carried single or multiple CpG dinucleotides either hyper- or hypomethylated, while 23 genes carried both hyper- and hypomethylated CpG dinucleotides (Tables S1, S2, and S7 in Supplementary Material). The number of differentially methylated CpG dinucleotides per gene was correlated with gene density and length suggesting a direct role on these CpG dinucleotides in controlling the activity of the genes they map within (Wilcoxon Rank-Sum Test *p*-value = 0.047).

### Pathway Analysis Comparing All PCOS Offspring with Controls

Ingenuity pathway analysis enlisted 908 of the 918 unique genes carrying differentially methylated CpG dinucleotides and clustered 794 of the 908 genes into 21 gene networks (Table S8 in Supplementary Material). Because the vast majority of unique genes (720 unique genes, or 91% of the total 794 genes distributed across all networks) resided in networks 1–10, we focused our analysis on these 10 networks (Table S9 in Supplementary Material).

**Figure 2:**
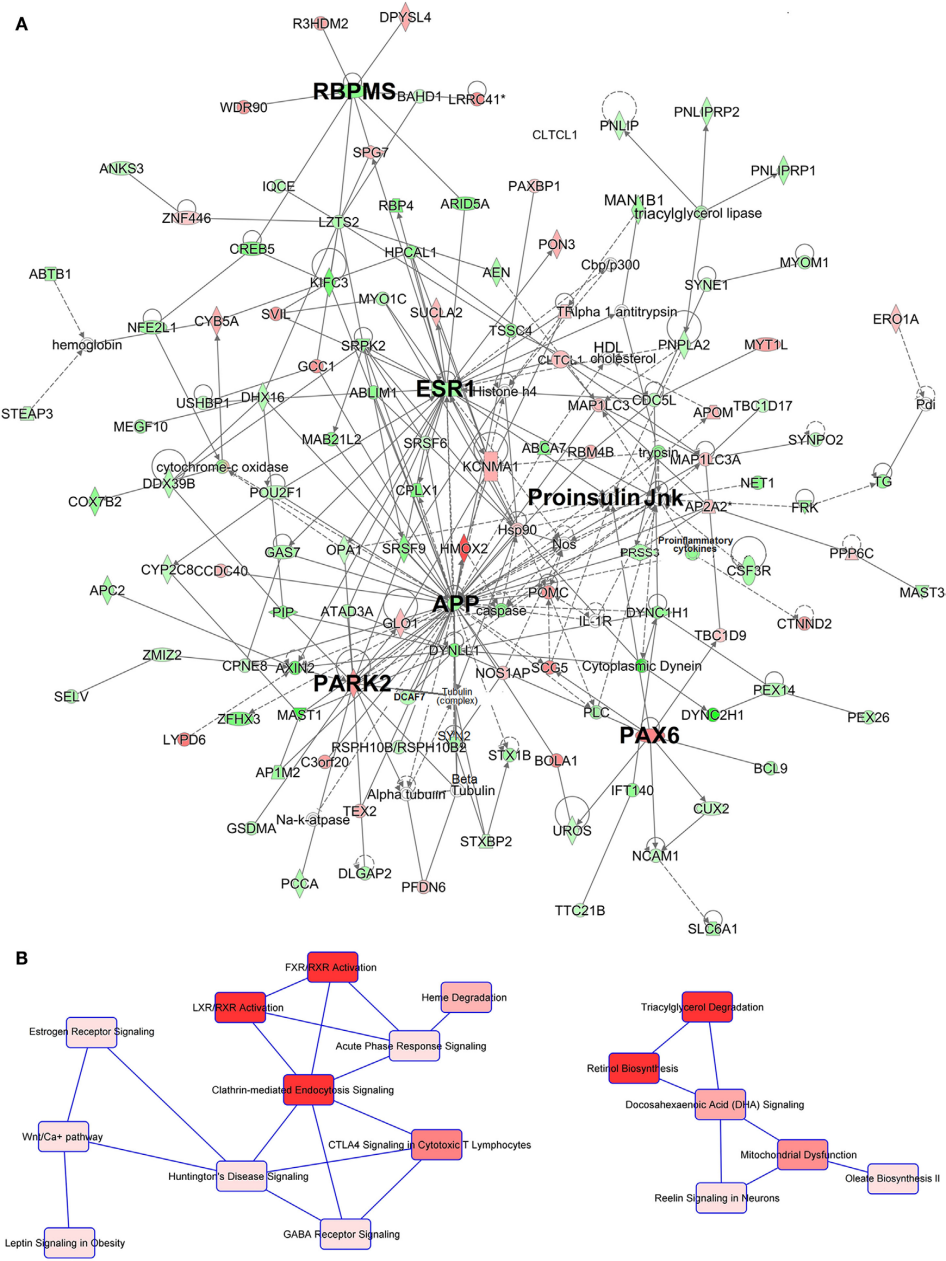
Ingenuity pathway analysis (IPA) network and canonical pathway analysis for the genes included in network **(A)**. Depiction of network 1. The main nodes are identified with bigger bolded font (see Table 2 for details). Gene/domain symbol shading: green, hypomethylated; red, hypermethylated; white, no differential methylation. For hyper- and hypomethylated genes/domains, red/green gradient relates to the methylation level. **(B)** Depiction of the two main canonical pathway webs of network 1 highlighted by IPA. The first (left) appears principally devoted to the regulation of the trafficking of hormones, proteins, cholesterol, fatty acid, and glucose with an important hormonal involvement. The second (right) is instead centered on the lipid metabolism and mitochondrial functioning regulation. Canonical pathway red symbol gradient relates to the *p*-value for the likelihood of the association between the differentially methylated genes in our experiment and the pathway. The smaller the *p*-value, the darker the red shading and the stronger the association. For a detailed explanation of molecule shapes and relationship types, see http://qiagen.force.com/KnowledgeBase/articles/Basic_Technical_Q_A/Legend.

Table 2 summarizes the main findings of the IPA analysis (see also Tables S8 and S9 in Supplementary Material). The highest scored network by IPA was Network 1, the “Carbohydrate Metabolism, Lipid Metabolism, Small Molecule Biochemistry” network (Figure 2A; Table S8 in Supplementary Material). This network included seven main nodes, five of which highlight three features of this network, namely hormonal regulation (*ESR1*), metabolic rate control through the control of the mitochondrial activity (*APP, PARK2*), and glucose metabolism [Proinsulin domain (*INS* is the focus gene), *PAX6*].

**Table 2:** Summary of the ingenuity pathway analysis (IPA) network and pathway analysis.

IPA Network						Canonical pathways		Top upstream regulator
Title		Main characteristics^a^	Main nodes	Other genes^b^^,^^c^	Figure	Main pathways	Figure	
1.	Carbohydrate metabolism, lipid metabolism, small molecule biochemistry	Genes (%): 117 (84)	↓ *APP*	↓ *ABCA7*	2 A	Clathrin-mediated endocytosis	2B	*FGF1*
		Hypo (%): 80 (68)	↓ *ESR1*	↓ *BCL9*		FXR/RXR		
		Hyper (%): 37 (32)	↑ *PARK2*	↓ *PCCA*		LXR/RXR		
			↑ *PAX6*	↓ *RBP4*		Estrogen receptor signaling		
			↓ *RBPMS*	↓ *SRPK2*		Triacylglycerol degradation		
			– Proinsulin	↓ *UROS*		Retinol biosynthesis		
			– Jnk			Docosahexaenoic acid signaling		
						Mitochondrial dysfunction		

2.	Cancer, organismal injury and abnormalities, gastrointestinal disease	Gene (%): 103 (74)	↓↑ Gprc^d^	↑ *BMP8B*	S1A	G-Coupled receptor signaling	S2A	*LEP*
		Hypo (%): 69 (67)	↑ *MOV10*	↓ *LRBA*		Insulin receptor signaling		
		Hyper (%): 34 (33)		↓ *RNF144B*				
				↑ *SYNJ2*				

3.	Cancer, gastrointestinal disease, organismal injury and abnormalities	Gene (%): 86 (61)	↓ Creb (*CREB5*)	↓ *ASGR1*	S1B	D-myo-inositol-5-phosphate metabolism	S2B	*ZDHHC7*
		Hypo (%): 61 (71)	↓ Mapk (*MAP3K6*)	↓ *DLG4*		Calcium signaling		
		Hyper (%): 25 (29)	↑ Pka (*PRKAR1B*)	↓ *DUSP8*		Dopamine-DARPP32 feedback in cAMP signaling		
			↓↑ Pkc(s) (*PRKCZ*^e^, *PRKCH*^f^)	↓ *RGS12*				
			↓ *FASN*					

4.	Cellular development, cellular growth and proliferation, hematological system development and function	Gene (%): 78 (56)	↓ Tlr (*TLR5*)	↓ *MRPL11*	S1C	PKCθ signaling in T lymphocytes	S2C	*INFA*
		Hypo (%): 56 (72)	↓↑ HLA-DR (*HLA-DRA, HLA-DRB1*)			CD28 signaling in T helper cells		
		Hyper (%): 22 (28)	↓ NFκB (*RELA*^f^)			Cdc42 signaling		
			↓ *ZBTB16*			Protein kinase A signaling		
			↓ *IRAK3*			Type I diabetes mellitus signaling		
			↓ *IRF4*					

5.	Cellular growth and proliferation, tissue development, cardiovascular system development and function	Gene (%): 74 (53)	↓ *SMAD3*	↓ *FN1*	S1D	Thrombin signaling	S2D	*SHH*
		Hypo (%): 52 (70)	↓ *RELA*	↓ *VIM*		GNRH signaling		
		Hyper (%): 22 (30)	↓ *FN1*			Dendritic cell maturation		
			↓ Estrogen Receptor (*ESR1*^g^)			NFκB signaling		
			– Mmp			HMGB1 signaling		
			– Vegf			Androgen signaling		
			– Cyclin-D					
			↑ P38 MAPK (*MAP3K6*^h^)					

6.	Neurological disease, posttranslational modification, carbohydrate metabolism	Gene (%): 68 (49)	↓ *DLG4*	↓ *ABCA7*	S1E	Factors promoting cardiogenesis in vertebrates	S2E	*TGFB1*
		Hypo (%): 44 (65)	– *SMARCA*	↑ *BMP8B*		Calcium signaling		
		Hyper (%): 24 (35)	– *MYC*			Chondroitin/dermatan/heparan sulfate biosynthesis		
			– *UBC*			NANOG		
			– *NXF1*			Role of NOTCH in embryonic stem cells		
			– *TGFB1*					
			– *IL10RA*					

7.	Cellular development, cellular growth and proliferation, embryonic development	Gene (%): 66 (47)	– *CUL3*	↓ *BCL9*	S1F	Superpathway of cholesterol biosynthesis	S2E	*TP73*
		Hypo (%): 39 (59)	– *TCF3*	↓ *LRBA*		Retinol biosynthesis		
		Hyper (%): 27 (41)	– *ELAVL1*	↓ *RNF144B*		Wnt/β-catenin signaling		
			– *FOS*			Fatty acid biosynthesis initiation II		
			– *TP73*			Chondroitin/dermatan/heparan sulfate biosynthesis		
			↓ *TCF21*			eNOS		

8.	Cancer, organismal injury and abnormalities, gastrointestinal disease	Gene (%): 61 (44)	– *HNF4A*	↓ *ASGR1*	S1G	Thyroid hormone metabolism	S2E	*HNF4A*
		Hypo (%): 41 (67)	– Histone h3	↓ *DUSP8*		Serotonin degradation		
		Hyper (%): 20 (33)	– *EED*	↓ *MRPL11*		Melatonin degradation I		
			– *IL1B*	↓ *RBP4*		Nicotine degradation II and III		
				↓ *UROS*		Role of OCT4 in embryonic stem cells		

9.	Auditory and vestibular system development and function, embryonic development, organ development	Gene (%): 62 (45)	– *CTNNB1*	↓ *ASL*	S1H	Clathrin-mediated endocytosis	S2E	*CBX5*
		Hypo (%): 41 (66)	– *TP53*	↓ *BCL9*		FXR/RXR		
		Hyper (%): 21 (34)	– *RAC1*	↓ *PCCA*				
			↓ *FN1*	↓ *RBP4*				
			– *STAT5A*	↓ *RGS12*				
				↑ *SYNJ2*				

10.	Amino acid metabolism, cancer, organismal injury and abnormalities	Gene (%): 60 (44)	↓ *ESR1*	↓ *ASGR1*	S1I	Urea cycle	S2E	*LOXL2*
		Hypo (%): 38 (63)	– *TNF*	↓ *VIM*		Citrulline nitric oxide cycle		
		Hyper (%): 22 (37)	– *ERBB2*			Arginine biosynthesis IV		
			– *EGFR*			Estrogen receptor signaling		
			↓ *VIM*			nNOS Signaling in neurons		
			– *FBXO6*					

*^a^Genes (%) = number and percent of the genes of each network belonging to the list of genes carrying differentially methylated CpG dinucleotides from this study (percentage calculated over the 140 genes used by IPA to populate each network); Hypo (%) = number and percentage of genes carrying hypomethylated CpG dinucleotides (percentage calculated over the per-network total number of genes carrying differentially methylated CpG dinucleotides from this study); Hyper (%) = number and percentage of genes carrying hypermethylated CpG dinucleotides (percentage calculated over the per-network total number of genes carrying differentially methylated CpG dinucleotides from this study)*.

*^b^Genes are reported in capital letters italicized; domains are reported as per the IPA notation. The symbols in the same rows of the genes/domains refer to: ↓ = hypomethylated, ↑ = hypermethylated, – = not scored by this study. For the differentially methylated domains in parenthesis the key differentially methylated genes are reported. Double symbols for some domains are reported because different members of the same domain show opposite differential methylation. In these cases, the size of the symbols reports the prevalent methylation status*.

**Figure 3:**
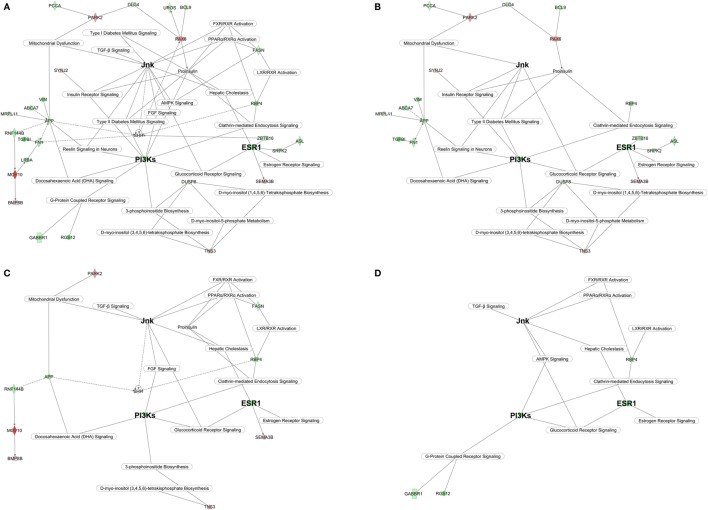
Polycystic ovary syndrome (PCOS) superpathway. **(A)** Full superpathway. **(B)** Glucotoxic PCOS component; **(C)** Lipotoxic PCOS component; **(D)** Inflammatory PCOS component. The main genes/domain common to the three components are identified with bigger bolded font. The design of the PCOS superpathway has been conducted in ingenuity pathway analysis by linking statistically significant key genes and canonical pathway highlighted by networks 1–10. Gene/domain symbol shading: green, hypomethylated; red, hypermethylated; white, no differential methylation. For hyper- and hypomethylated genes/domains, red/green gradient relates to the methylation level.

*^c^It includes the main differentially methylated genes contemporaneously appearing in different networks together with key genes for the canonical pathways listed and for the PCOS superpathway of Figure 3*.

*^d^This domain includes a large list of genes belonging to Network 2 as detailed here following. Hypomethylated: ADRA1B, CCKBR, CHRM1, CHRM5, GABBR1, GIPR, GPR108, GPR37L1, HRH1, LGR5, MAS1L, MC3R, S1PR4, TAS1R3. Hypermethylated: GPR15, GPR151, GPR19, GRM5, LHCGR, RXFP3, and TSHR*.

*^e^Methylation status derived by IPA from Network 11 (data not shown)*.

*^f^Methylation status derived by IPA from Network 5*.

*^g^Methylation status derived by IPA from Network 1*.

*^h^Methylation status derived by IPA from Network 3*.

The canonical pathways analysis of network 1 (Figure 2B) revealed two main pathway webs. The first includes Clathrin-Mediated Endocytosis, the key cellular entry port for metabolites hormones and proteins, the FXR/RXR, and the LXR/RXR activation pathways which regulate bile acid trafficking and cholesterol, fatty acid, and glucose homeostasis, respectively, and involve the activity of INS. This web connects with the Estrogen Receptor Signaling pathway centered on the activity of the estrogen receptor *ESR1* which interfaces with the Wnt pathway (Figure 2B, left). The second web involves lipid metabolism and regulation of mitochondrial function determined by genes *APP* and *PARK2* within network 1. This web is centered on the canonical pathways for the Triacylglycerol Degradation, Retinol Biosynthesis, Docosahexaenoic Acid (DHA) Signaling and Mitochondrial Dysfunction (Figure 2B, right). Interestingly, the IPA upstream regulator analysis also highlighted the effect of the Triacylglycerol Degradation pathway on the activity of the *FGF1* growth factor, a known player for hepatic and peripheral insulin resistance.

Network 2 (Figure S1A in Supplementary Material) was dominated by the G-Protein Coupled Receptor Signaling pathway that directly interfaces with the Insulin Receptor Signaling pathway and other signaling pathways involved in inflammatory and regulatory metabolic functions (Figure S2A in Supplementary Material). These functions intersect with the activity of *MOV10*, a putative helicase gene related to the Phosphoinositide 3-Kinase (PI3K) family.

Network 3 (Figure S1B in Supplementary Material) highlights strong influence on lipid metabolism and neurotransmitter signaling. Network 3 included different forms of the d-myo-inositol Biosynthesis, key pathways for phosphatidylinositol metabolism and neurotransmitter signaling (Figure S2B in Supplementary Material).

Networks 4 and 5 (Figures S1C and S1D in Supplementary Material) feature nodes involved in cardiovascular system development and function. Specifically, Network 4 includes pathways related to inflammatory responses (Figure S2C in Supplementary Material) with upstream analysis showing modification of activity of interferon alpha, a key pro-inflammatory cytokine. Network 5 is populated by pathways with important links to cardiovascular system development (Figure S2D in Supplementary Material). The canonical pathway webs of both network 4 and 5 were linked to Type I and II diabetes pathways, and network 5 linked with hepatic functions. Network 5 canonical pathways also included the Androgen Signaling pathway. The IPA upstream regulator analysis also pointed at differential effects on the regulation of the peptidase *SHH*, a key gene for the embryo development programing that is tightly linked to cholesterol metabolism.

Networks 6–10 (Figures S1E–S1I in Supplementary Material) highlight the role of the glycosaminoglycan family of signaling carbohydrates regulating the activity of the transcription factor *TGFB1* as highlighted by the IPA upstream regulator analysis. Additionally, differentially regulated pathways showed a role for control of amino acid biosynthesis centered on glutamate signaling and operated by the Urea Cycle, Arginine Biosynthesis and Citrulline biosynthesis pathways (Figure S2E in Supplementary Material). Of relevance is the significant scoring for the Thyroid Hormone Metabolism and the Estrogen Receptor Signaling pathways (Figure S2E in Supplementary Material). Among other relevant pathways are those that link the activity of networks 6–10 with those of networks 1–5 (Table 2) including Cholesterol Biosynthesis and Processing (*ABCA7, ASGR1*), FXR/RXR (*RBP4*) and LXR/RXR (*RBP4*); amino acids, fatty acids and glycogen metabolism (*FASN, PCCA*); estrogen receptor activity (*ESR1*); mitochondrial dysfunction (*APP*, Jnk domain, *PARK2*); G-Protein Coupled Receptors; Retinol pathways; and the PI3K family (*PIK3R5, PIK3R6*). These pathways highlight involvement of networks 6–10 in embryonic growth regulation suggested by co-existence with developmental pathways such as NANOG, NOTCH, eNOS, nNOS and OCT4 (Figure S2E in Supplementary Material).

### Gender-Specific Methylation Analysis

Due to the gender imbalance between the PCOS and control offspring (Table 1) initial analysis included all patients and controls thereby excluding all gender-specific differentially methylated CpG dinucleotides. However, to ascertain whether gender differences in epigenetic reprogramming existed during pregnancy a subsidiary gender-specific methylation analysis was also performed. In the female PCOS offspring, most hypomethylated pathways were associated with both lipid metabolism and carbohydrate/glucose metabolism. In addition, numerous inflammation related pathways (e.g., “lymphocyte activation,” “regulation of cytokine secretion,” and “positive regulation of cell adhesion”) were also significantly hypomethylated (i.e., possibly upregulated). In contrast, in the male PCOS offspring, only glucose metabolism and insulin signaling pathways were hypomethylated (i.e., possibly upregulated) while the lipid metabolism pathways were not differentially methylated.

As in the females, several, albeit significantly fewer, inflammation related pathways were also hypomethylated in the male PCOS offspring (“B cell differentiation,” “lymphocyte differentiation,” “cell activation during immune response”), these data suggest a more pronounced effect on lipid metabolism and inflammation in female than in male PCOS offspring.

## Discussion

Polycystic ovary syndrome is a heterogeneous syndrome characterized by progressive development of reproductive, metabolic, endocrine, and cardiovascular risk factor dysfunctions (5–7). Although antecedent features may be detected in infancy and childhood [e.g., increased anti-Müllerian hormone (AMH) levels], PCOS emerges as a distinct clinical entity in peri-puberty and evolves into its mature clinical form in late adolescence or early adulthood (6). Animal studies suggest that during pregnancy the maternal PCOS intrauterine environment affects the epigenetic programming of the fetus and replicates PCOS endocrine and metabolic dysfunctions in the progeny (15). To the best of our knowledge, our study is the first to explore whether, and if so, the nature and magnitude of the PCOS intrauterine environment affects human fetal epigenetic gene network programming.

Here, we showed differential DNA methylation in UCB from offspring of infertile women with PCOS compared to offspring of women without PCOS. These reveal unique epigenetic signatures which may play a role in the underlying systemic dysfunctions of PCOS, namely glucotoxicity, lipotoxicity and chronic systemic inflammation. Moreover, the data pinpoint the role of key non-reproductive organs that participate in the pathophysiology of the syndrome including among others the brain, liver, kidney, muscle, and pancreas.

Ingenuity Pathway Analysis identified 10 networks which taken together suggest the existence of a putative PCOS “superpathway” linking key gene networks with major canonical biologic pathways (Figure 3A). The putative PCOS “superpathway” may generate the endocrine and metabolic phenotype of PCOS (Figures 3B–D).

Our data suggest that the primary initiating feature of the PCOS epigenetic signature is the gene coding for the estrogen receptor α, *ESR1* (network 1) (Figure 3). The upregulation of *ESR1 in utero* may be the basis for the burst of metabolic syndrome dysfunctions occurring during the peri-pubertal transition into adolescence and beyond. The *ESR1* driven signaling cascade upregulates PI3Ks (18, 19) (network 2) shaping the PCOS glucotoxic environment (Figure 3A).

Our data, while preliminary and in need of confirmation, suggest the importance of certain pathways in the epigenetic imprinting of PCOS *in utero*. PI3Ks modulate oxidative stress and insulin resistance via the DHA Signaling (network 1) (20) and the Insulin Receptor Signaling (network 2) pathways and the Jnk domain (network 1) and is highlighted by significant scoring of the Type II Diabetes Mellitus Signaling pathway (network 5). PI3Ks also modulate glucose uptake and storage (21) in the brain through the Reelin Signaling in Neurons pathway (network 1) which has been shown to affect feeding behaviors (22) and promote insulin resistance (23). In addition it activates the hypomethylated Amyloid Beta Precursor Protein (*APP*) gene (network 1). Elevated *APP* also activates the Jnk domain impairing mitochondrial metabolism and increasing oxidative stress rates (24, 25), which promote additional system wide but particularly brain insulin resistance (26). In the current study *PARK2* is hypermethylated suggesting limited mitochondrial autophagy and turnover of damaged mitochondria which may further intensify brain insulin resistance and oxidative stress.

Phosphatidylinositol phosphate lipids (PIPs) are second messengers regulated by PI3Ks and controlled by myo-inositol metabolism (network 3). In diabetics, depletion of intracellular myo-inositol (27, 28) has been observed in insulin sensitive tissues such as liver, muscle, fat, and kidney (29, 30) affecting local and systemic glucose uptake and disposal dynamics. The pancreatic involvement in the glucotoxic PCOS epigenetic signature is highlighted by hypermethylation of the *PAX6* gene (network 1). *PAX6* is a transcription factor crucial for pancreatic beta-cell differentiation and survival. Elimination of *PAX6* activity, an epigenetic result of uterine artery occlusion induces diabetes and a dramatic reduction in the number of beta cells producing insulin in the intrauterine growth restricted rodent model (31). Alterations of aminoacid metabolism in the PCOS signature (network 10) correlate with reports associating type II diabetes with elevated plasma levels of the aminoacids/aminoacid precursors citrulline and arginine.

*ESR1*, the main signatory initiating the glucotoxic PCOS signature, by altering PIPs, DHA and inositol biosynthesis, also leads to the second major metabolic feature of PCOS, lipotoxicity (Figure 3C). In the liver *ESR1* modulates hepatic cholestasis (network 5), FXR/RXR, and LXR/RXR (network 1) pathways that control bile acid turnover, regulate lipid and cholesterol metabolism leading to dyslipidemia. Specifically, estrogens, *via ESR1*, promote dyslipidemia by inhibiting bile acid secretion and interfere with hepatocyte uptake (32, 33). The strong hypermethylation of *MOV10* (network 2) is also highly relevant since the downregulation of this gene is associated with altered lipid metabolism (34).

The IPA upstream regulator analysis also suggests inhibition of the *FGF1* growth factor (network 1). Downregulation of the fibroblast growth factor family results in increased hepatic steatosis, worsening of the serum lipid profiles, and insulin resistance (35). Additionally, the lipotoxic PCOS epigenetic factors affecting the brain may be mediated by upregulation of the upstream regulator sonic hedgehog (*SHH*) peptidase (network 5) a gene that plays a critical role in brain cholesterol metabolism (36).

Finally, the hypomethylation of transforming growth factor beta 1 (*TGFB1*) (network 6) suggests a role in the PCOS phenotype. *TGFB1* participates in the linkage of lipotoxicity, glucotoxicity, and chronic systemic inflammation (Figure 3D).

To the best of our knowledge, this is the first study to investigate and identify the induction of unique fetal epigenetic reprogramming in progeny of PCOS mothers. By contrasting global DNA methylation patterns in the UCB of babies born to PCOS and non-PCOS mothers, a putative “PCOS signature” emerges which reflects assembly of 10 significantly upregulated inter-active gene networks linked to major canonical biologic pathways. While substantially expanding the knowledge of the epigenetic genesis of PCOS, taken together, such reprogramming explains the induction of the glucotoxicity, lipotoxicity, and systemic inflammatory state underlying the PCOS clinical phenotype. As such it is consistent with and confirms existing genetic and epigenetic evidence (Table 3) (37–43). Furthermore, should these preliminary results receive independent confirmation by examination of larger cohorts, important advances in the understanding of the pathogenesis and management of PCOS will emerge. Specifically its transgenerational transmission, and with the possibility of early discovery of susceptibility, strategies may be designed for prevention, moderation and/or reversal well before the progressive dysfunction and disease burdens of PCOS are entrained.

**Table 3:** Overlapping findings between the existing literature and the work presented in this study^a^.

Literature		This study		
Gene(s), reference	Description	Gene/domain	Description/interaction	Network/pathway^b^
**a. Genetics**				

*CYP11A, CYP17* (37–39)	Cytochrome P450 family members 11A and 17	*CYP2C8, CYP2F1*	Cytochrome P450 family members 2C8, 2F1	Network: 1, 6
				Pathway: FXR/RXR Activation
*TNF* (37–39)	Tumor necrosis factor	*TNFRSF1A, TNFRSF1B*	Tumor necrosis factor receptor superfamily members 1A and 1B	Network: 5
				Pathway: Type I and Type II Diabetes Mellitus
*PPARG* (37–39)	Peroxisome proliferator-activated receptor gamma	PPAR/RXRA	Heterodimer regulating transcription	Network: 1, 3, 5, 6, 9, 10
				Pathway: PPAR/RXRA Activation
*RAB5B* (40)	RAS oncogene family member 5B	*RAB19*	RAS oncogene family member 19	Network: 9
				Pathway: –
*ZNF557* (40)	Zinc finger protein member 557	*ZNF71, ZNF331, ZNF446, ZNF586*	Zinc finger protein members 71, 331, 446, 586	Network: 1, 3, 4, 6, 7, 8
				Pathway: –
*STXBP1* (41)	Syntaxin binding protein 1	*STXBP2*	Syntaxin binding protein 2	Network: 1
				Pathway: –
*LAMA1* (44, 45)	Laminin chain alpha 1	*LAMA5, LAMB2*	Laminin chain alpha 5, beta 2	Network: 9
				Pathway: CDK5 signaling

**b. Epigenetics**				

*CYP19A1* (42, 43)	Cytochrome P450 family member 19A1	*CYP2C8, CYP2F1*	Cytochrome P450 family members 2C8, 2F1	Network: 1, 6
				Pathway: FXR/RXR Activation
*HOXA10* (42, 43)	Homeobox member A10	*HOXA7*	Homeobox member A7	Network: 3
				Pathway: –
*IGF2BP2* (42, 43)	Insulin-like growth factor 2 mRNA binding protein 2	*IGF2BP2*	Insulin-like growth factor 2 mRNA binding protein 2	Network: 3
				Pathway: –
*LHCGR* (42, 43)	Luteinizing hormone/choriogonadotropin receptor	*LHCGR*	Luteinizing hormone/choriogonadotropin receptor	Network: 2
				Pathway: G-Protein Coupled Receptor Signaling
*PDE4D* (42, 43)	Phosphodiesterase family member 4D	*PDE4A, PDE4D, PDE11A*	Phosphodiesterase family members 4A, 4D, 11A	Network: 4
				Pathway: G-Protein Coupled Receptor Signaling
*SLC7A8* (42, 43)	Solute carrier family member 8	*SLC2A9, SLC6A1, SLC6A4, SLC7A4, SLC7A8, SLC12A5, SLC14A1, SLC16A3, SLC22A18, SLC25A23, SLC27A1, SLC29A1, SLC43A2*	Solute Carrier Family members 2A9, 6A1, 6A4, 7A4, 7A8, 12A5, 14A1, 16A3, 22A18, 25A23, 27A1, 29A1, 43A2	Network: 1, 2, 3, 5, 6, 7, 8, 10
				Pathway: –

*^a^For those comparisons where literature findings refer to genes that are part of large gene families for which we identified differential methylation on some gene members, we carried out a confirmatory Ingenuity Pathway Analysis (IPA) (data not shown). We analyzed all genes of the same family reported in the table by running independent IPA analyses by gene family. By using the more stringent setup that generates 35-items networks, which is ideal for small gene lists as networks get generated only if genes have direct and more relevant connections, each gene family from our list only generated one 35-item network. This test supports our approach that is meant at showing that alterations of the methylation status of the genes presented here, and belonging to the same gene families, affect gene networks that have been shown to work together to carry out specific cellular functions*.

*^b^Pathways are reported when univocal or when known*.

The main limitations of this preliminary, exploratory study are the limited number of patients (*n* = 6) and the imbalance of progeny gender between cases and controls (although gender-specific analysis demonstrated quantitative not qualitative differences). However, even though our dataset was small, it was a carefully selected homogenous and well characterized cohort of PCOS patients, all showing anovulatory infertility, polycystic ovarian morphology, and increased AMH profile of the Rotterdam diagnostic category. Most importantly, our cohort did not have hyperandrogenism eliminating the possibility of epigenetic changes driven by high androgen levels.

In conclusion, our study suggests that the maternal PCOS intrauterine environment affects the epigenetic programming of the developing embryo by inducing important marks in genomic regions characterizing the PCOS phenotype. This PCOS epigenetic “signature” may be responsible for perpetuating the maternal endocrine and metabolic dysfunctions in the progeny. If confirmed, our data supports the notion of the intrauterine origin of adult disease. Additional larger and more detailed studies are needed to confirm these provocative preliminary findings.

## Ethics Statement

The study was done in accordance with the principles expressed in the Helsinki declaration, and that the study was approved by the Icahn School of Medicine at Mount Sinai Institutional Review Board (IRB). The Materials and Methods section of the paper provides details about the protocol number. About the consent procedure, written consent was obtained from every study subject. Patients were recruited during visits to RMA (Reproductive Medicine Associates) of New York, or at initial prenatal visits at Mount Sinai Hospital. Providers notified the appropriate member of the study team of the potential subject. The provider asked the potential subject if they were interested in participating in the study. If they were, the provider gave them the study team’s contact information, or asked the potential subject if they would like to speak with the study team at that time. Once the subject was identified and permission obtained to contact the subject, a member of the study team authorized to obtain informed consent was called on-site to approach the potential subject about the research study. Potential subjects were provided both verbally and in writing information regarding the study. Potential subjects were given the opportunity to ask questions regarding the study and/or enrollment, and were provided with a copy of the consent to read.

No massive sequencing methodologies have been used for the completion of the aims of this study. The microarray analysis of CpG dinucleotides differs from whole-genome or whole-exome sequencing as the results (methylation levels at each CpG dinucleotide analyzed) would not show any incidental genetic findings. Our IRB reviewed this issue and approved the study.

## Author Contributions

Wrote the manuscript: LL and SS. Carried out genetic data analysis: LL. Contributed to the acquisition of the clinical data and samples: AC. Contributed to the writing of the manuscript: SH. Carried out the DNA methylation data analysis: ZY and WZ. Designed the study: YT and NK. Agreed with manuscript results and conclusions: LL, SS, AC, SH, ZY, WZ, YT, and NK. Made critical revisions and approved final version: LL. All the authors revised and approved the final manuscript and agreed to be accountable for the content of the work.

## Conflict of Interest Statement

The authors declare that the research was conducted in the absence of any commercial or financial relationships that could be construed as a potential conflict of interest.
