# Supplementary material for: Intrauterine Reprogramming of the Polycystic Ovary Syndrome: Evidence from a Pilot Study of Cord Blood Global Methylation Analysis
Source: Front Endocrinol (Lausanne). 2017 Dec 18;8:352. doi: 10.3389/fendo.2017.00352 (PMC5741701; doi:10.3389/fendo.2017.00352)
Supplement: Supplementary file 2 [file Image_1.PDF]

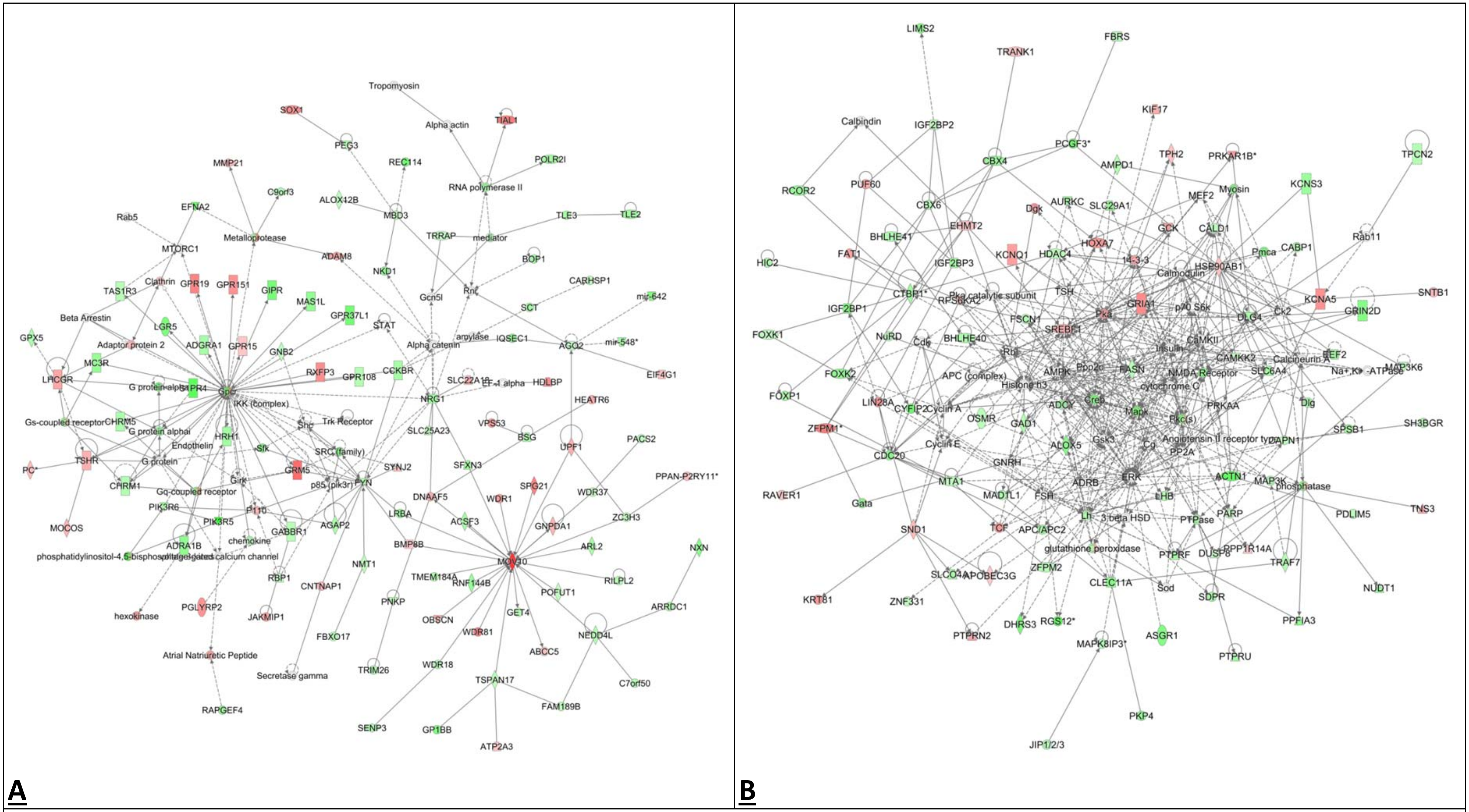


See legend page 5

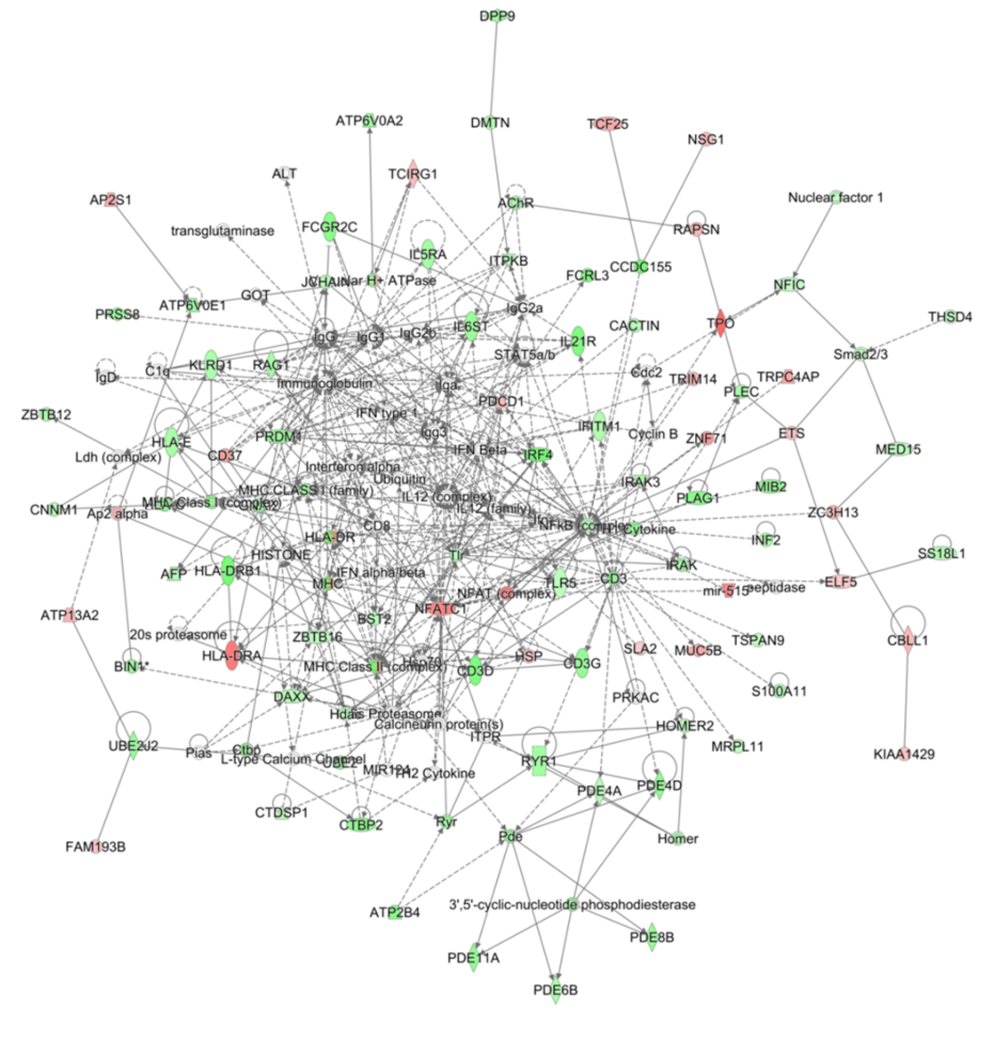


**C**

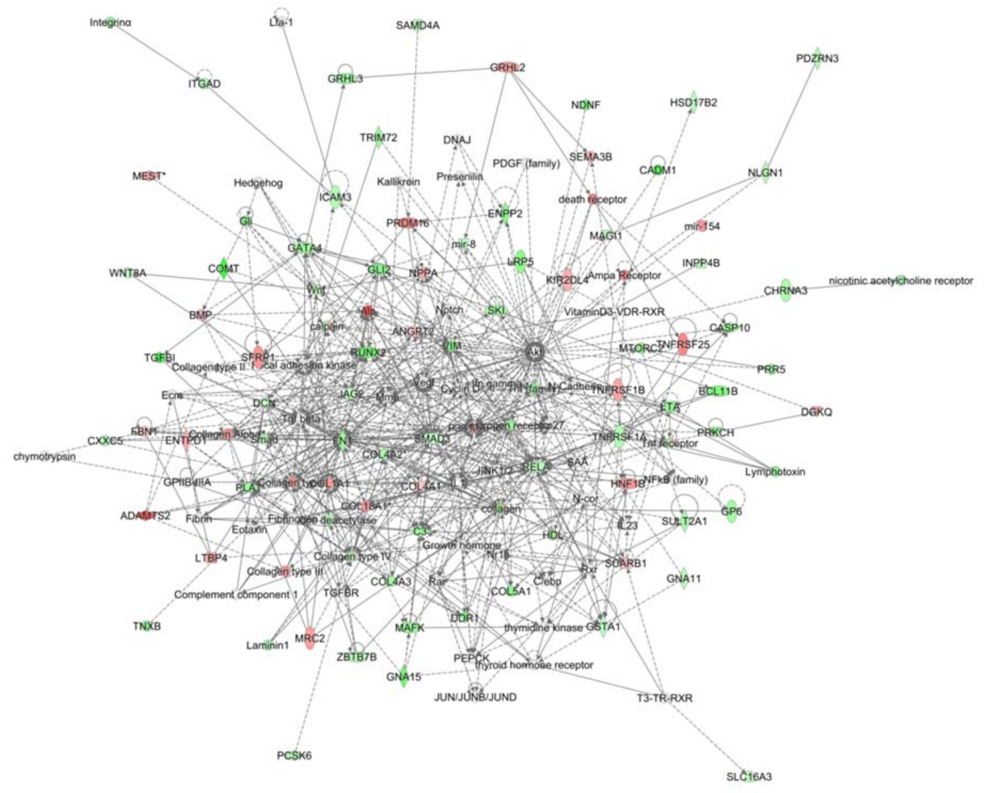


**D**

See legend page 5

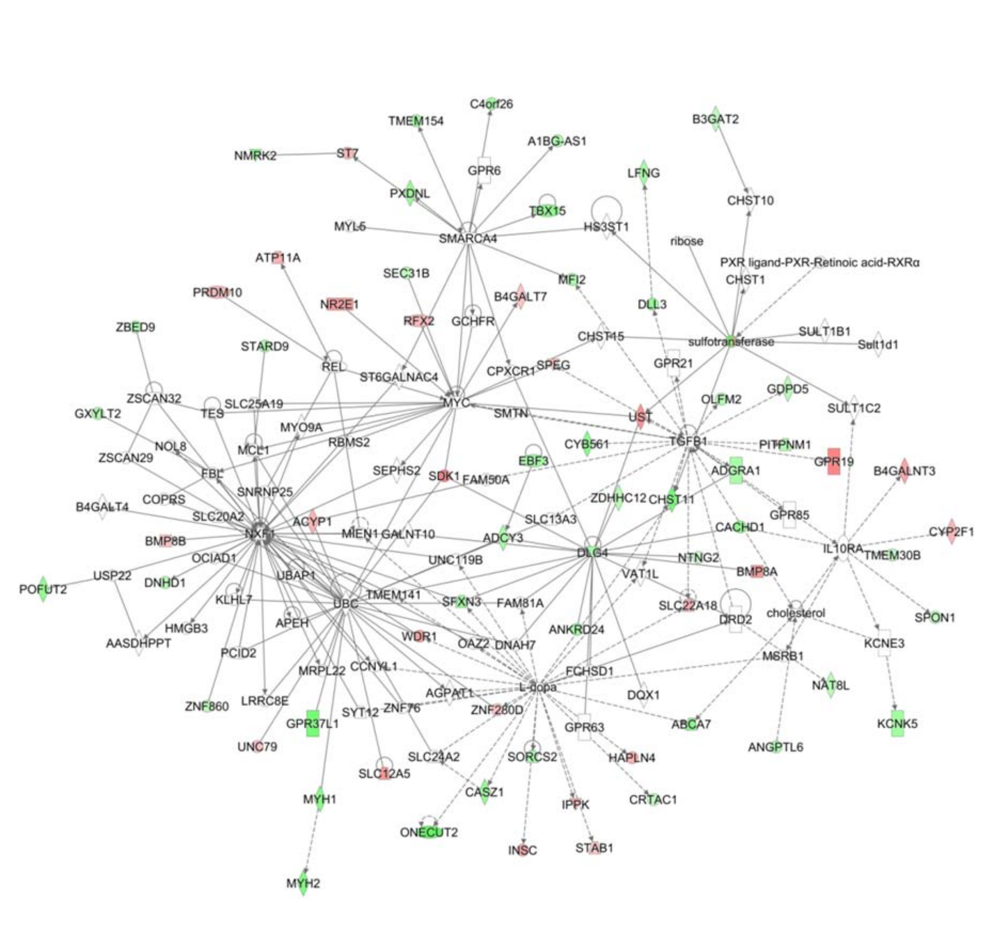


**E**

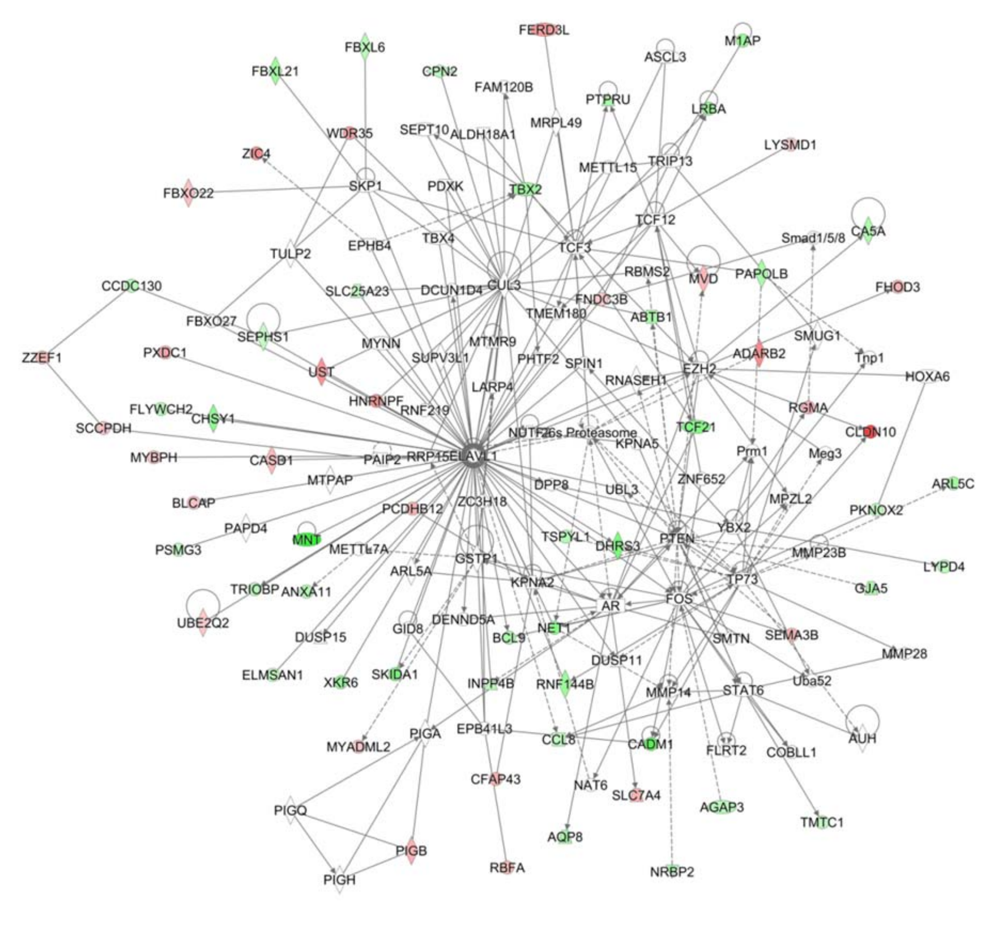


**F**

See legend page 5

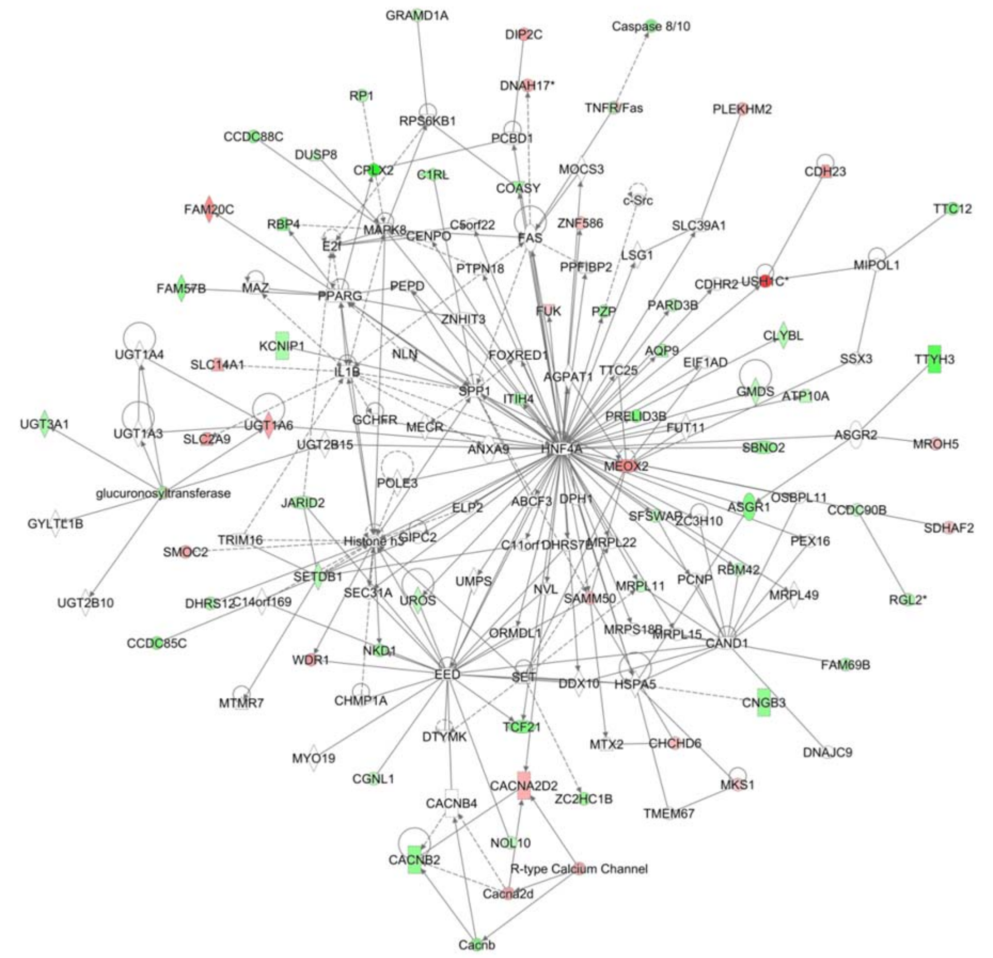


**G**

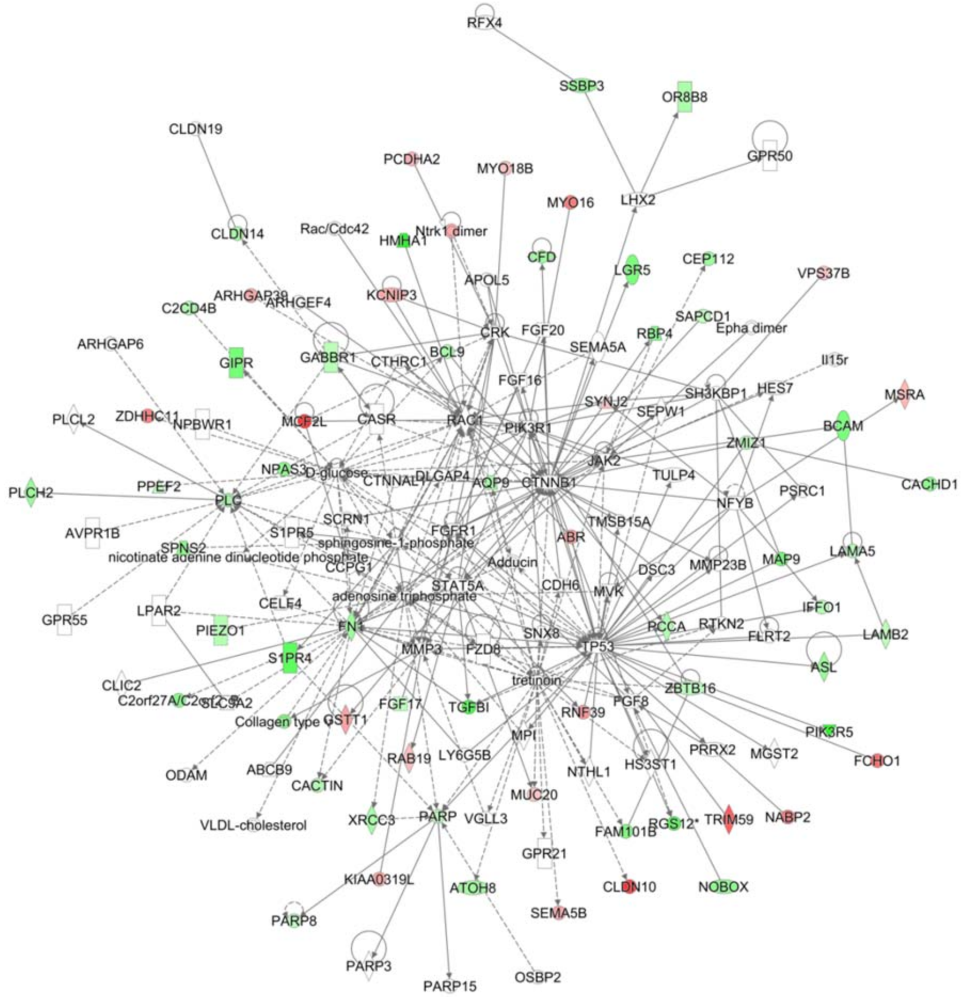


**H**

See legend page 5

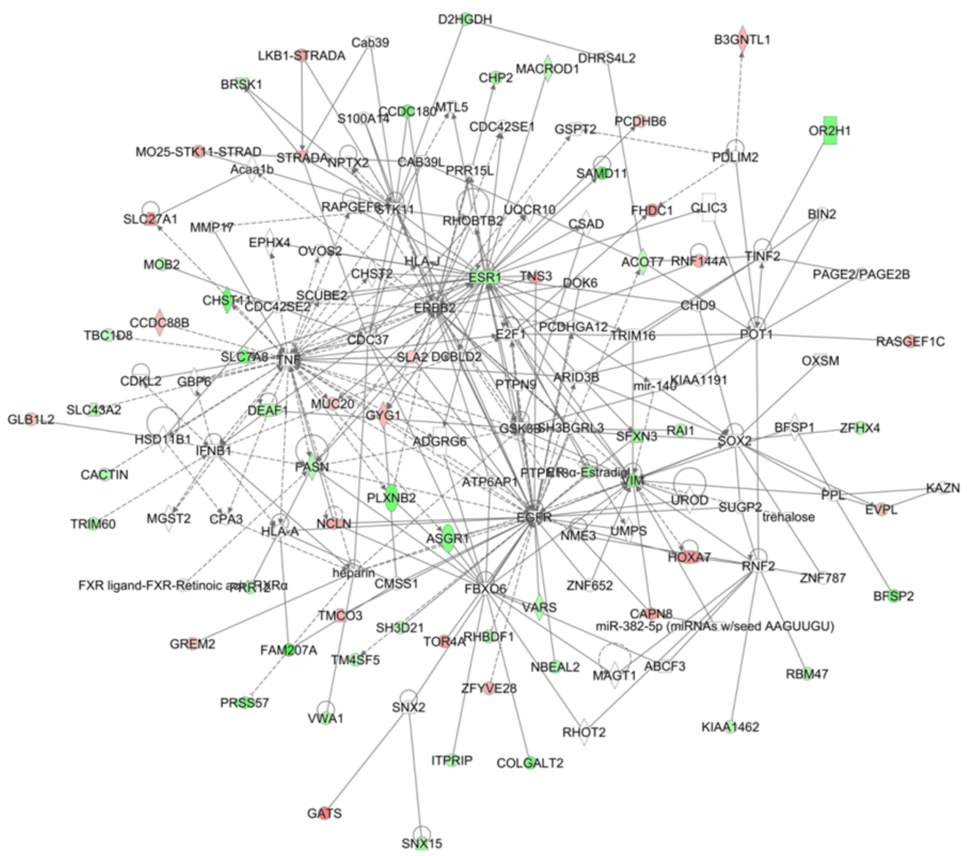


**I**

Figure S1. IPA networks 2 (A), 3 (B), 4 (C), 5 (D), 6 (E), 7 (F), 8 (G), 9 (H) and 10 (I). See Table S11 for details. Gene/domain symbol shading: green = hypomethylated; red = hypermethylated; white = no differential methylation. For hyper- and hypomethylated genes/domains, red/green gradient relates to the methylation level.

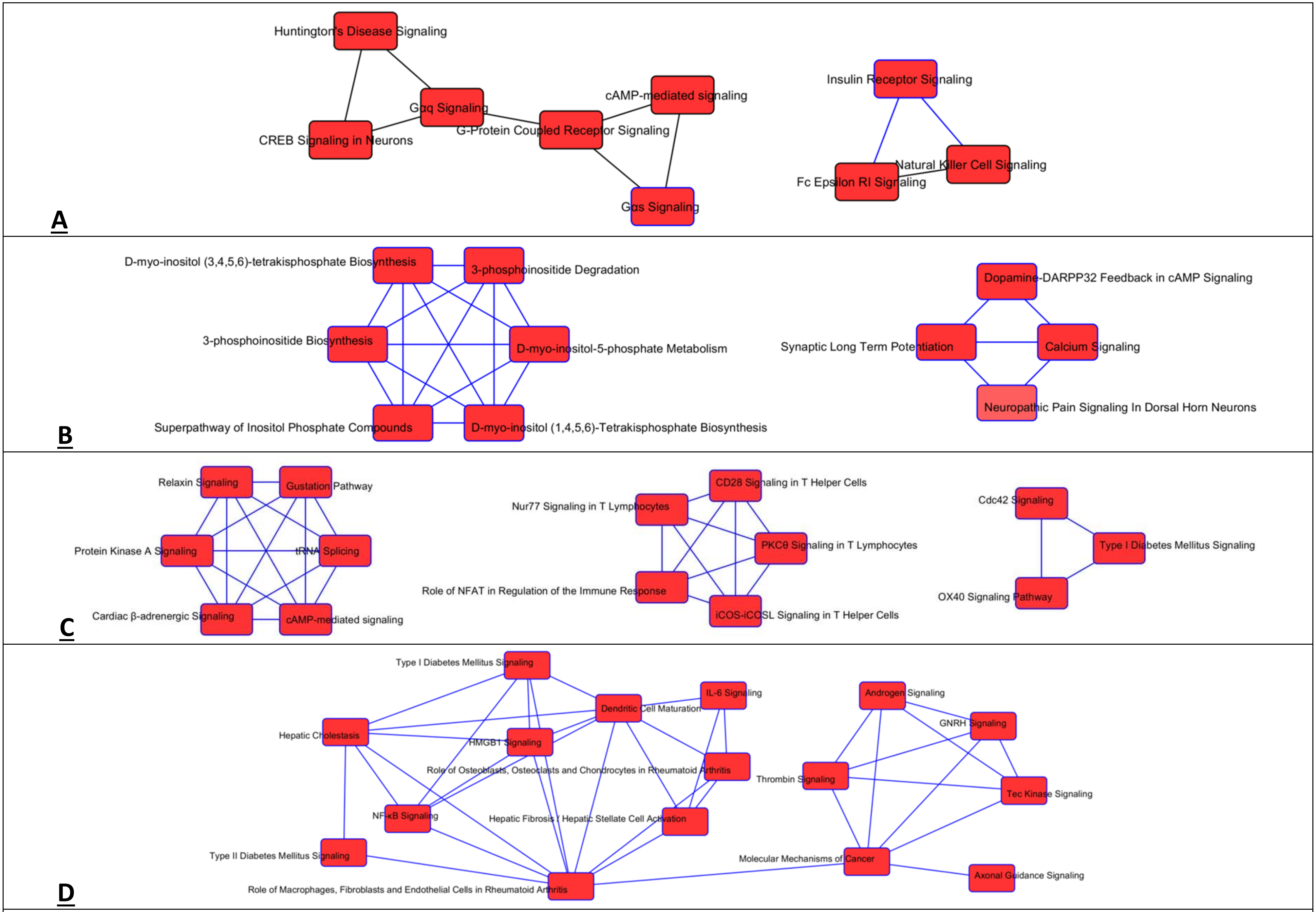


See legend page 7

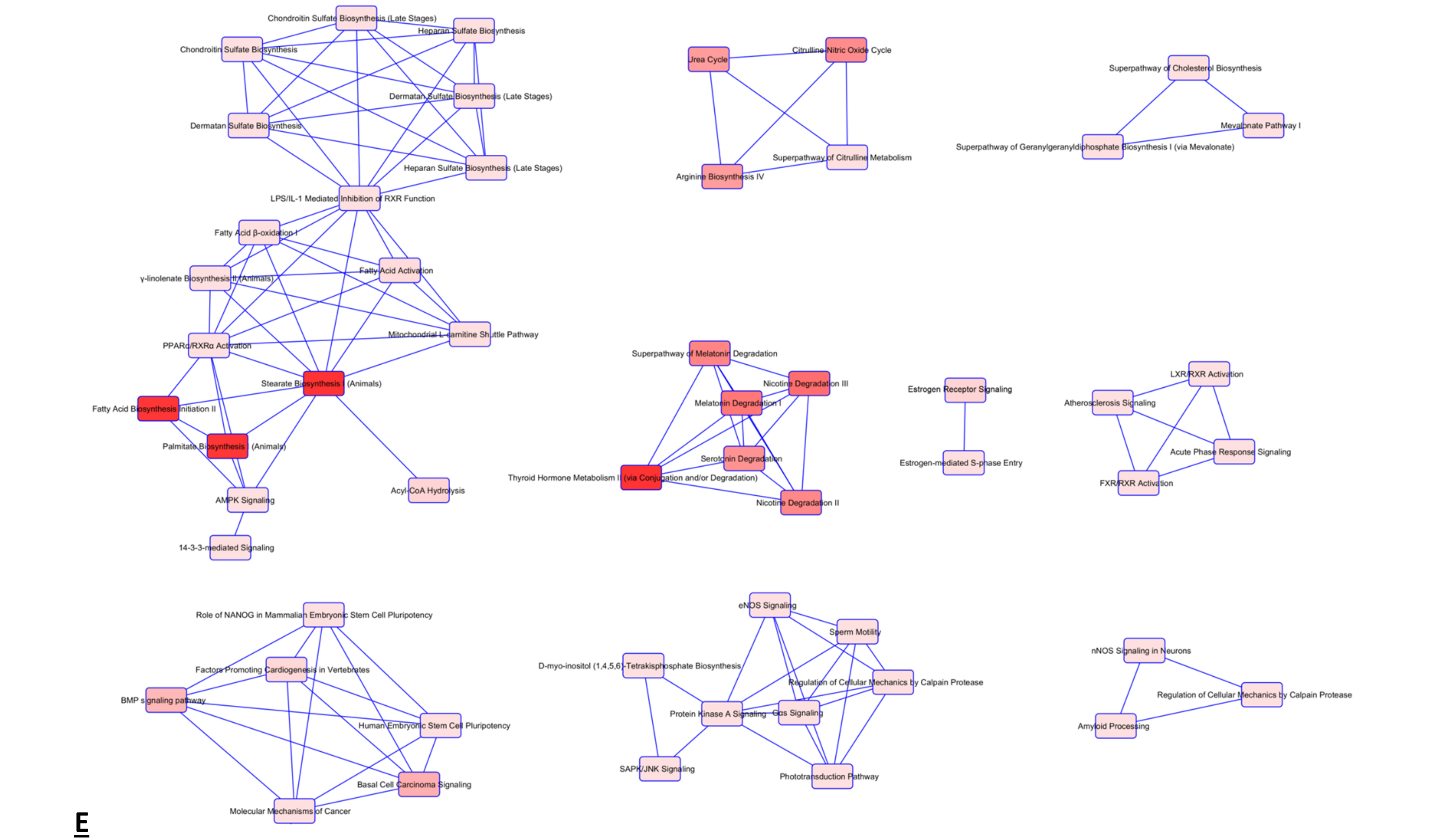


Figure S2. Depiction of the main canonical pathways scored by the genes of network 2 (A), 3 (B), 4 (C), 5 (D) and summary of the main canonical pathways scored by the genes of networks 6 to 10 (E). Canonical pathway red symbol gradient relates to the p-value for the likelihood of the association between the differentially methylated genes in our experiment and the pathway. The smaller the p-value the darker the red shading and the stronger the association.
